# Supplementary figures and images for: Investigating the added value of biomarkers compared with self-reported smoking in predicting future e-cigarette use: Evidence from a longitudinal UK cohort study
Source: PLoS One. 2020 Jul 14;15(7):e0235629. doi: 10.1371/journal.pone.0235629 (PMC7360042; doi:10.1371/journal.pone.0235629)

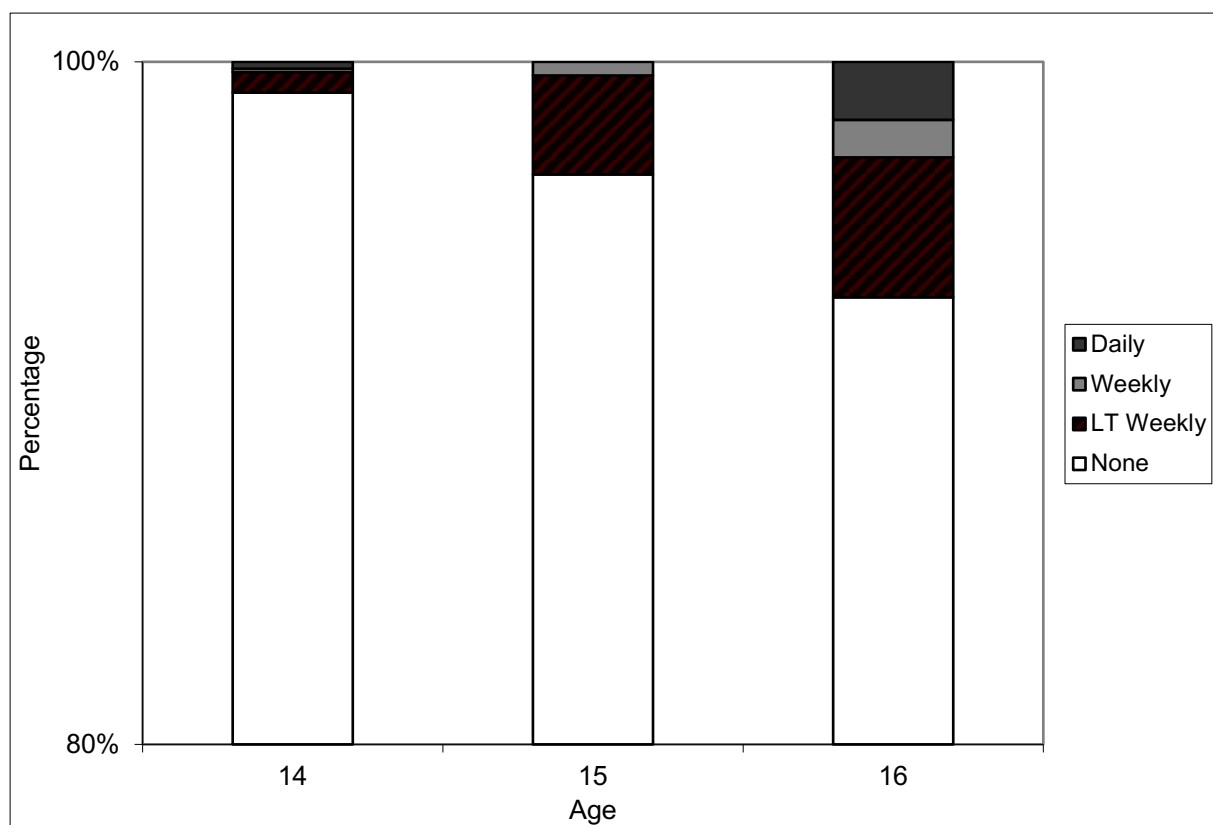

**S1 Fig. Probability of being a daily, weekly, long-term weekly or never smoker in latent class 1.**

Supplement: S1 Fig — (PDF) [file pone.0235629.s001.pdf]

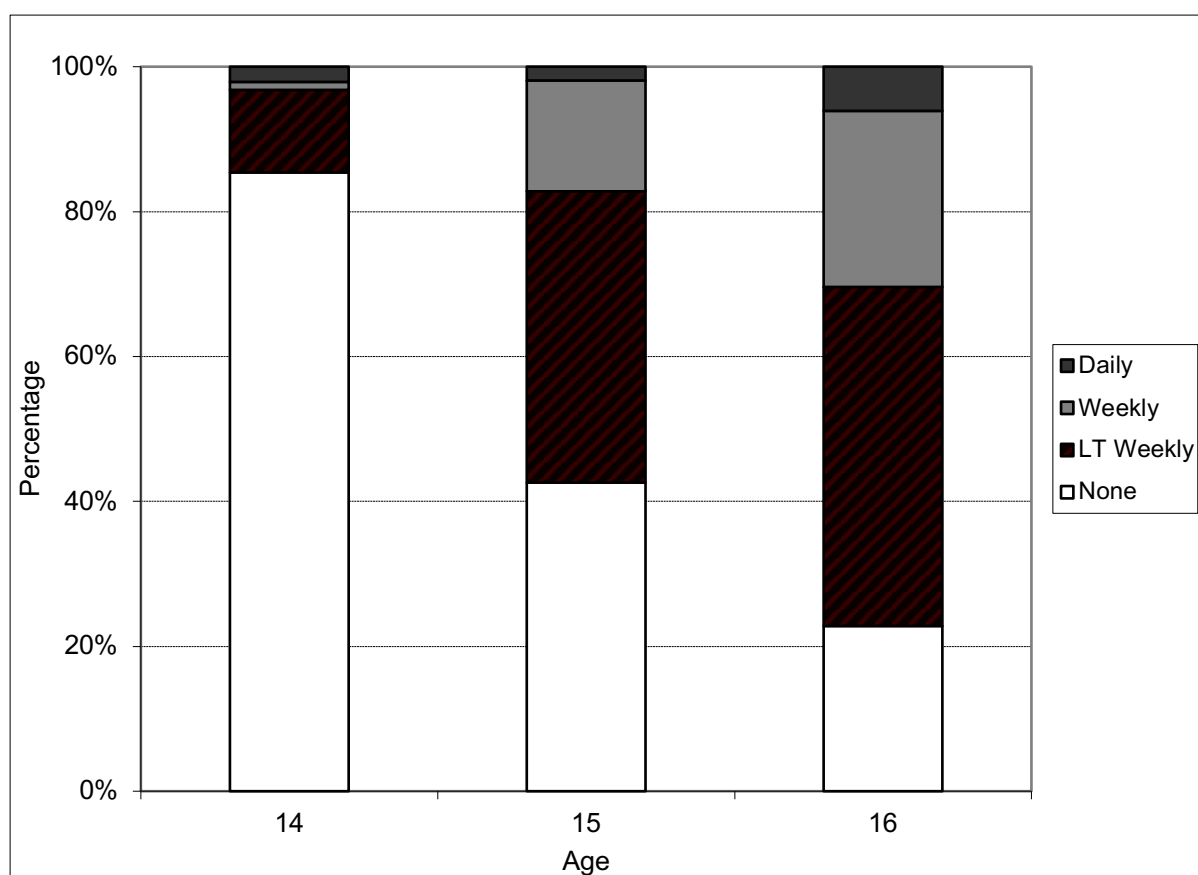

**S2 Fig. Probability of being a daily, weekly, long-term weekly or never smoker in latent class 2.**

Supplement: S2 Fig — (PDF) [file pone.0235629.s002.pdf]

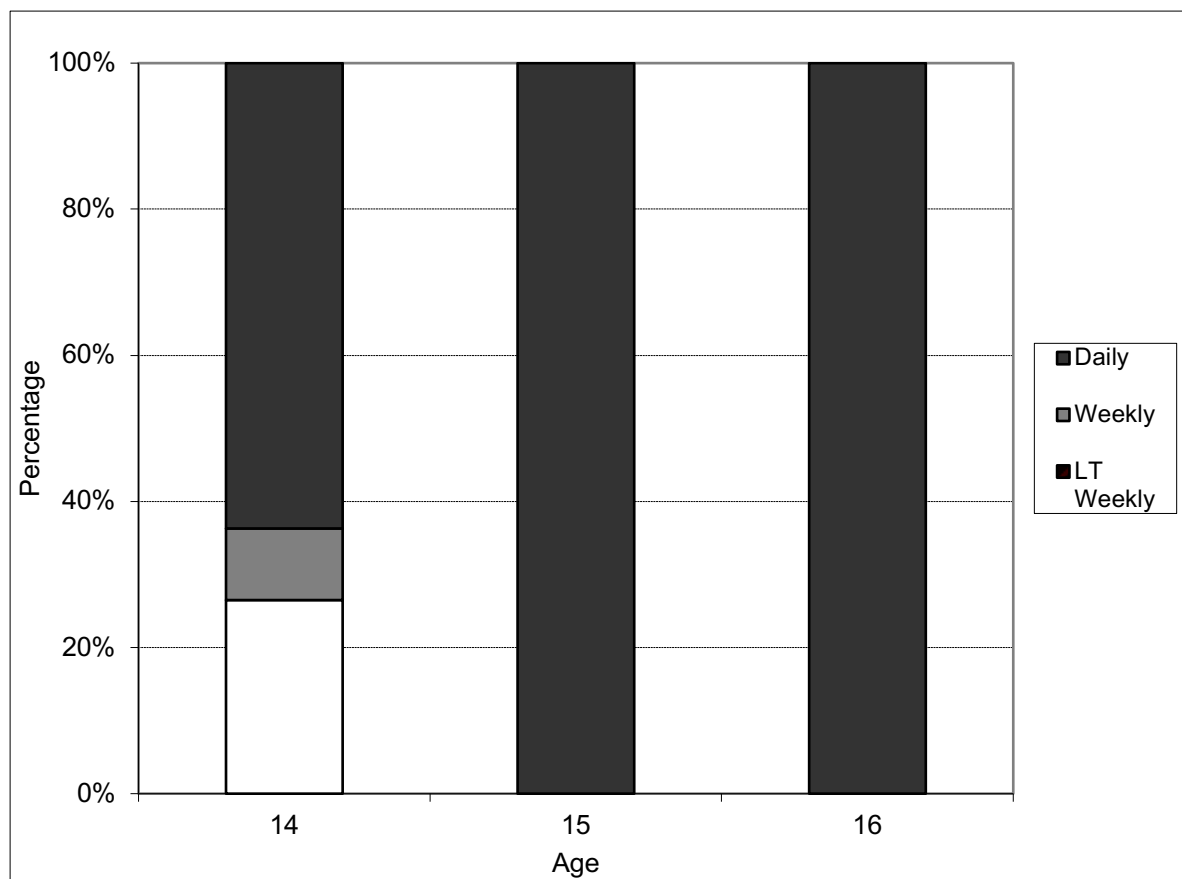

**S3 Fig. Probability of being a daily, weekly, long-term weekly or never smoker in latent class 3.**

Supplement: S3 Fig — (PDF) [file pone.0235629.s003.pdf]

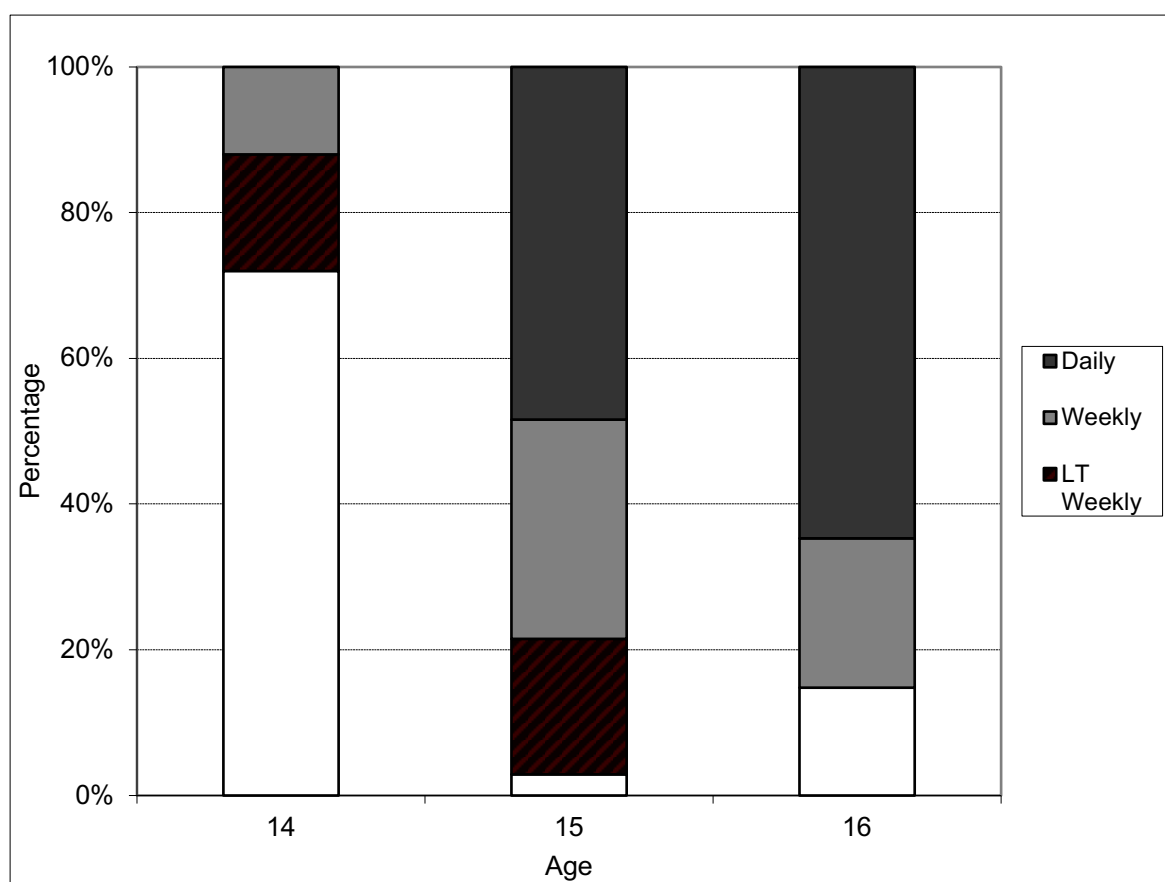

**S4 Fig. Probability of being a daily, weekly, long-term weekly or never smoker in latent class 4.**

Supplement: S4 Fig — (PDF) [file pone.0235629.s004.pdf]
