# Supplementary material for: Investigating the added value of biomarkers compared with self-reported smoking in predicting future e-cigarette use: Evidence from a longitudinal UK cohort study
Source: PLoS One. 2020 Jul 14;15(7):e0235629. doi: 10.1371/journal.pone.0235629 (PMC7360042; doi:10.1371/journal.pone.0235629)
Supplement: S2 Table — Reference group = no exposure; OR = odds ratio; 95% CI = 95% confidence interval. Cotinine was treated as a categorical variable in these analyses. Active exposure is defined as cotinine levels exceeding 10 ng/ml in blood samples. The basic model (model 1) was adjusted for age and sex. Model 2 was additionally adjusted for socioeconomic status, BMI, alcohol and cannabis use. Models 4a-4c were as model 3 and additionally adjusted for various self-reported measures of smoking and the difference in age between the cotinine measure and the self-report. Model 4a adjusted for ever smoking at age 16. Model 4b alternatively adjusted for number of cigarettes smoked by age 16. Model 4c alternatively adjusted for active smoking (daily/weekly) at age 16. Model 4d was as model 3 and adjusted for classes of smoking transitions; early onset regular smokers, late onset regular smokers, never smokers and experimenters categorised using data from 14 to 16. (PDF) [file pone.0235629.s006.pdf]

S2 Table. Associations of cotinine at 15 years and ever use of e-cigarettes at 22 years (N=1,194) including cannabis use as a covariate.

Model	Cotinine		
	Active		
	OR	95% CI	*p*-value
1	10.47	4.88, 22.46	<.001
2	4.67	2.06, 10.54	<.001
3	4.23	1.86, 9.66	<.001
4a	3.89	1.71, 8.88	.001
4b	2.16	0.90, 5.23	.087
4c	2.31	0.95, 5.61	.066
4d	2.35	0.84, 6.58	.103

Reference group = no exposure; OR = odds ratio; 95% CI = 95% confidence interval. Cotinine was treated as a categorical variable in these analyses. Active exposure is defined as cotinine levels exceeding 10 ng/ml in blood samples. The basic model (model 1) was adjusted for age and sex. Model 2 was additionally adjusted for socioeconomic status, BMI, alcohol and cannabis use. Models 4a-4c were as model 3 and additionally adjusted for various self-reported measures of smoking and the difference in age between the cotinine measure and the self-report. Model 4a adjusted for ever smoking at age 16. Model 4b alternatively adjusted for number of cigarettes smoked by age 16. Model 4c alternatively adjusted for active smoking (daily/weekly) at age 16. Model 4d was as model 3 and adjusted for classes of smoking transitions; early onset regular smokers, late onset regular smokers, never smokers and experimenters categorised using data from 14 to 16.
