# Supplementary material for: Investigating the added value of biomarkers compared with self-reported smoking in predicting future e-cigarette use: Evidence from a longitudinal UK cohort study
Source: PLoS One. 2020 Jul 14;15(7):e0235629. doi: 10.1371/journal.pone.0235629 (PMC7360042; doi:10.1371/journal.pone.0235629)
Supplement: S3 Table — Reference group = no exposure; OR = odds ratio; 95% CI = 95% confidence interval. Cotinine was treated as a categorical variable in these analyses. Active exposure is defined as cotinine levels exceeding 10 ng/ml in blood samples. The basic model (model 1) was adjusted for age and sex. Model 2 was additionally adjusted for socioeconomic status, BMI and alcohol. Model 3 was additionally adjusted for passive smoke exposure (maternal smoking at 12 years). Models 4a-4c were as model 3 and additionally adjusted for self-reported measures of smoking and the difference in age between the self-report and cotinine measures. Model 4a adjusted for ever smoking at age 16. Model 4b alternatively adjusted for number of cigarettes smoked by age 16. Model 4c alternatively adjusted for active smoking (daily/weekly) at age 16. Model 4d was as model 3 and adjusted for classes of smoking transitions; early onset regular smokers, late onset regular smokers, never smokers and experimenters categorised using data from 14 to 16. (PDF) [file pone.0235629.s007.pdf]

S3 Table. Associations of cotinine at 15 years and ever use of e-cigarettes at 22 years restricting to those with age differences below 18 months (n=870).

Model	Cotinine		
	Active		
	OR	95% CI	*p*-value
1	8.60	3.66, 20.23	<.001
2	7.05	2.94, 16.93	<.001
3	6.31	2.60, 15.31	<.001
4a	4.29	1.75, 10.53	.001
4b	1.92	0.72, 5.15	.19
4c	2.88	1.08, 7.65	.03
4d	2.18	0.66, 7.20	.20

Reference group = no exposure; OR = odds ratio; 95% CI = 95% confidence interval. Cotinine was treated as a categorical variable in these analyses. Active exposure is defined as cotinine levels exceeding 10 ng/ml in blood samples. The basic model (model 1) was adjusted for age and sex. Model 2 was additionally adjusted for socioeconomic status, BMI and alcohol. Model 3 was additionally adjusted for passive smoke exposure (maternal smoking at 12 years). Models 4a-4c were as model 3 and additionally adjusted for self-reported measures of smoking and the difference in age between the self-report and cotinine measures. Model 4a adjusted for ever smoking at age 16. Model 4b alternatively adjusted for number of cigarettes smoked by age 16. Model 4c alternatively adjusted for active smoking (daily/weekly) at age 16. Model 4d was as model 3 and adjusted for classes of smoking transitions; early onset regular smokers, late onset regular smokers, never smokers and experimenters categorised using data from 14 to 16.
