# Supplementary material for: Investigating the added value of biomarkers compared with self-reported smoking in predicting future e-cigarette use: Evidence from a longitudinal UK cohort study
Source: PLoS One. 2020 Jul 14;15(7):e0235629. doi: 10.1371/journal.pone.0235629 (PMC7360042; doi:10.1371/journal.pone.0235629)
Supplement: S1 File — (PDF) [file pone.0235629.s008.pdf]

S1 File. Parental education

Parental education was measured as follows: Degree, A-level, O-level, less than O-level. O levels were qualifications taken at age 16 which have been replaced by GCSEs [General Certificate of Secondary Education] in England, Wales and Northern Ireland. A-levels are exams taken at age 18 in these countries.
